# Whole-genome sequencing for every newborn in the UK: promise and practicalities

**DOI:** 10.1016/j.eclinm.2025.103387

**Published:** 2025-07-23

**Authors:** eClinicalMedicine

In June 2025, the UK National Health Service announced a plan to offer whole-genome sequencing to every newborn within the next decade, with a national rollout beginning in 2026. The move comes as part of the 10-Year Health Plan aiming to align health and growth objectives with a greater focus on proactive and preventive medicine. The £650 million initiative follows pilot programmes like the Generation Study by Genomics England, which showed that whole-genome sequencing could identify rare, treatable conditions in about one in every 200 babies. The landmark policy holds promise, aiming to catch disease earlier and reduce future burden on health and spending. However, it also carries with it some concerns regarding clinical utility, consent, data protection, and implementation.

Currently, newborns in the UK can have a heel-prick blood test at around 5 days old to screen for nine conditions, such as cystic fibrosis, sickle cell disease, and phenylketonuria, for which early treatment before symptoms arise has been shown to improve outcomes. In other countries, the number of diseases screened for varies: the USA tests for 63 disorders, whereas Australia tests for 32, and in countries such as China, the number varies by region or province. The UK's proposed national genome sequencing programme will be offered alongside traditional newborn screening using biochemical assays, and will use a blood sample from the umbilical cord to analyse each child's full DNA sequence, to screen for more than 200 rare but treatable conditions, such as spinal muscular atrophy, childhood cancers, neurodevelopmental disorders, severe immunodeficiencies, and cardiac abnormalities. Detecting this wider range of rare conditions would enable early treatment for many more patients, to prevent harm from delayed treatment and reduce health burden and mortality.

There are a few key reasons why this can be seen as a positive policy shift. Studies have shown that whole-genome sequencing can improve the diagnostic yield and provide timely refutation of false-positive results from traditional newborn screening for diseases such as cystic fibrosis. In a pilot study in China, whole-genome sequencing produced fewer false-positive results and identified more actionable pathogenic variants than routine newborn screening. Although genome sequencing is relatively expensive, with an estimated cost of £1000 per genome, it is becoming cheaper over time and early diagnoses could lead to reduced spending later in life on delayed or misdirected care. Given ongoing advances in technology and automation, rapid whole-genome sequencing could cost as little as $100 per genome at scale. Additionally, it could provide a platform for lifelong health care: an individual's genome can be reinterpreted and will be accessible to clinicians over time in response to new health issues or as part of family planning consultations, for example. Furthermore, offering sequencing to every newborn nationally will provide equal access and avoid it only being available to those able to pay for it or who reside in specific regions.

Such a novel policy also requires careful consideration of the clinical, ethical, and societal concerns. In a cohort study, although whole-genome sequencing yielded fewer false-positives than conventional blood-based newborn screening (0.04 vs 0.17%), it also gave more results of uncertain significance (0.90 vs 0.01%). Not all genomic variants are well understood, and results of uncertain significance could cause anxiety or lead to unnecessary intervention as the benefit of presymptomatic treatment is often unclear. Even among genetic variants labelled as pathogenic, penetrance (likelihood that a person carrying a particular variant will actually exhibit the associated trait or disease) might be low or context dependent. Therefore, in some cases, early diagnosis might be of dubious benefit, and risk of false positives cannot be excluded. Additionally, the presence of an abnormality could potentially have an impact on access to health or life insurance. Informed consent is also important to consider: newborns cannot provide consent, and parents would need to explicitly consent to long-term storage of the baby's genomic data, which could lead to affecting the individual later in life. Only a small portion of their genome will be screened for their direct benefit, and similar screenings could be performed using much more limited genetic data at a substantially lower cost. Therefore, if whole genomes are collected for newborn screening, there must be a clear and well justified reason for doing so. There are also concerns over data privacy and long-term use: a national genomic database, even one with strong safeguards, raises inevitable concerns about surveillance, misuse, and hacking risks. Protecting this type of data is challenging and potentially exploitable, and there is a risk that it could be improperly shared with third parties such as insurance companies, pharmaceutical firms, or law enforcement agencies. In 2023, genetic testing company 23 and Me had a breach that compromised the personal data of UK users, highlighting how important clear rules and regulations will be to protect such sensitive data.

Implementation of the policy might also face some hurdles. There is already a scarcity of genetic counsellors, and demand is likely to increase substantially as primary care providers might not yet have training in interpreting and communicating complex genetic results. A scalable approach to genomic education and support will be essential for accurately interpreting results and counselling families. In the USA, the BabySeq study evaluated the utility of newborn sequencing and found moderate benefits but greater risks than with traditional newborn screening and strong concerns over implementation from clinicians around clinical utility and potential discrimination based on genomic information. The value of early genomic information relies on its interpretation, delivery, and ongoing follow-up. Without supporting infrastructure, this could be a costly endeavour with uncertain results.

Careful implementation of whole-genome sequencing for newborns should involve various safeguards. It could be beneficial for parents to be able to opt in or out for different categories of findings––child-onset vs adult-onset conditions or carrier status, for example, to reduce potential family anxiety. The focus should be kept on actionable variants, aiming to avoid flagging results with low certainty or unclear relevance. Ongoing evaluation of outcomes, family experiences, and clinical utility will be essential to shape the programme to provide maximal benefit. And there will be a need for clear and transparent communication about what whole-genome sequencing involves, how the data will be used, and how the risks will be managed.

Whole-genome sequencing for newborns shows promise for identifying rare diseases earlier, reducing suffering and improving personalised care, but a responsible rollout will be crucial to ensure that families can benefit without additional burden.

